# Absence of cognitive symptoms in a 6-year-old male with post-traumatic increased intracranial pressure - A case report^☆^

**DOI:** 10.1016/j.amsu.2018.09.021

**Published:** 2018-09-25

**Authors:** Fadi Al Daoud, Anne Drolet, Chase Carto, Haben Debessai, Gul Sachwani Daswani

**Affiliations:** 1 Hurley Plaza, 7 B Trauma Services, Flint, MI, 48503, USA

**Keywords:** Trauma, Apnea, Bradycardia, Intracranial pressure, Midline shift, TBI, Traumatic Brain Injury, GCS, Glasgow Coma Scale, ICP, Intracranial Pressure, ICH, Intracranial Hemorrhages

## Abstract

**Introduction:**

Traumatic Brain Injuries (TBIs) can range from mild to severe, and may result in increased intracranial pressure (ICP). Increased ICP causes hallmark physical signs, such as diaphoresis, emesis, fixed pupils, and altered mental status. Monitoring the patient's score on the Glasgow Coma Scale (GCS) and cranial CT scans are routine measures used in clinical practice to monitor the development of a TBI.

**Presentation of the case:**

A 6-year-old male fell off his father's shoulders and subsequently presented to ED for suspected head trauma. He was transferred to our Level 1 Trauma Center after a head CT scan demonstrated a subdural hematoma. His GCS score remained 15. The next day he began to have episodes of apnea and desaturation. Further imaging indicated expansion of the hematoma with a 5mm midline shift. He remained consistently alert and a neurological exam revealed cranial nerves to be grossly intact. Increased ICP was reduced with several days of hypertonic saline treatment without surgical intervention.

**Discussion:**

TBIs can have long-lasting effects in pediatric patients and are typically assessed using both diagnostic imaging and clinical judgment. CT scans are used to assess for hematoma development, while loss of consciousness (LOC) and altered mental status are standard clinical diagnostic indicators of increased ICP. This patient remained alert with a GCS score of 15, although he had clinical signs of increased ICP including apnea and bradycardia with a midline shift confirmed on imaging.

**Conclusion:**

While GCS is an important prognostic indicator in TBI, patients should still be monitored to assure resolution of all symptoms.

## Highlights

• Pediatric TBI remains the leading cause of disability and death in children over the age of 1 year.
• Children may differ in their response to TBI.
• GCS and head CT scans are routine measures to monitor the development of a TBI.
• Increased ICP associated with TBI may resolve on medical treatment without surgical intervention.
• Some pediatric patients with increased ICP may remained alert with normal GCS.

## 1 Introduction

Traumatic brain injuries (TBI) are external physical insults or injuries to the head that may cause brain damage [1]. Pediatric patients have a further increased susceptibility to even mild TBI, specifically in the temporal lobe [2]. This leads to decreased neurocognitive functions later in life. TBI in the pediatric population can range from mild concussions to severe intracranial hemorrhages (ICH). This range in severity can prove difficult to diagnose when assessing a pediatric patient symptomatically. TBIs remain the leading cause of disability and death in children over the age of 1 [3]. Complications associated with TBIs are most commonly due to cerebral edema, expansion of an extra-axial hematoma, and/or increased intracranial pressure [3]. Early recognition and effective management in minimizing secondary brain injury due to intracranial hypertension, hypercarbia, hypoxia, systemic hypotension, and increased metabolic demand are key in improving the outcomes of children with TBI [4].

## 2 Presentation of the case

**Fig. 1:**
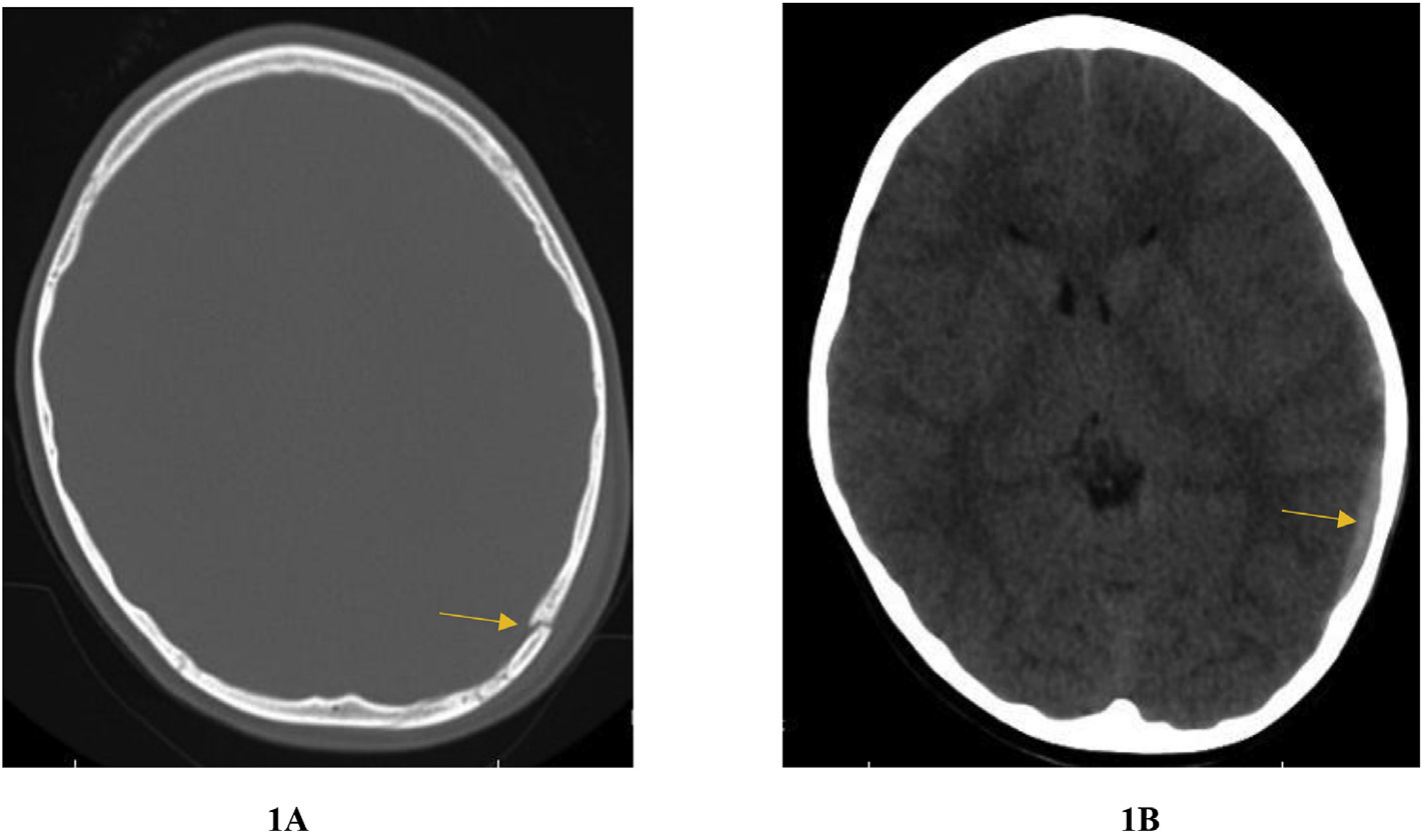
First CT scan showed a small, left-sided skull fracture (1A), and left-sided temporoparietal subdural (1B).

The patient is a 6-year-old male who presented with head trauma after falling from his father's’ shoulders onto the pavement. He was reported as being significantly diaphoretic with recurrent nausea and emesis. However, there was no loss of consciousness, shortness of breath, changes in vision, or pain in the extremities. A CT scan indicated a small, left-sided skull fracture and a 4 mm temporoparietal subdural hematoma (Fig. 1). The patient was then transferred to our Level 1 Trauma Center.

Upon presentation, his vital signs were the following: BP 78/43, T 37C oral, HR 87, RR 19, and SpO2 99% on room air. His physical exam was unremarkable, except for occipital tenderness to palpation. He was then admitted to the PICU for close monitoring. Because his GCS remained at 15 and cranial nerves were grossly intact, no further neuroimaging was warranted.

**Fig. 2:**
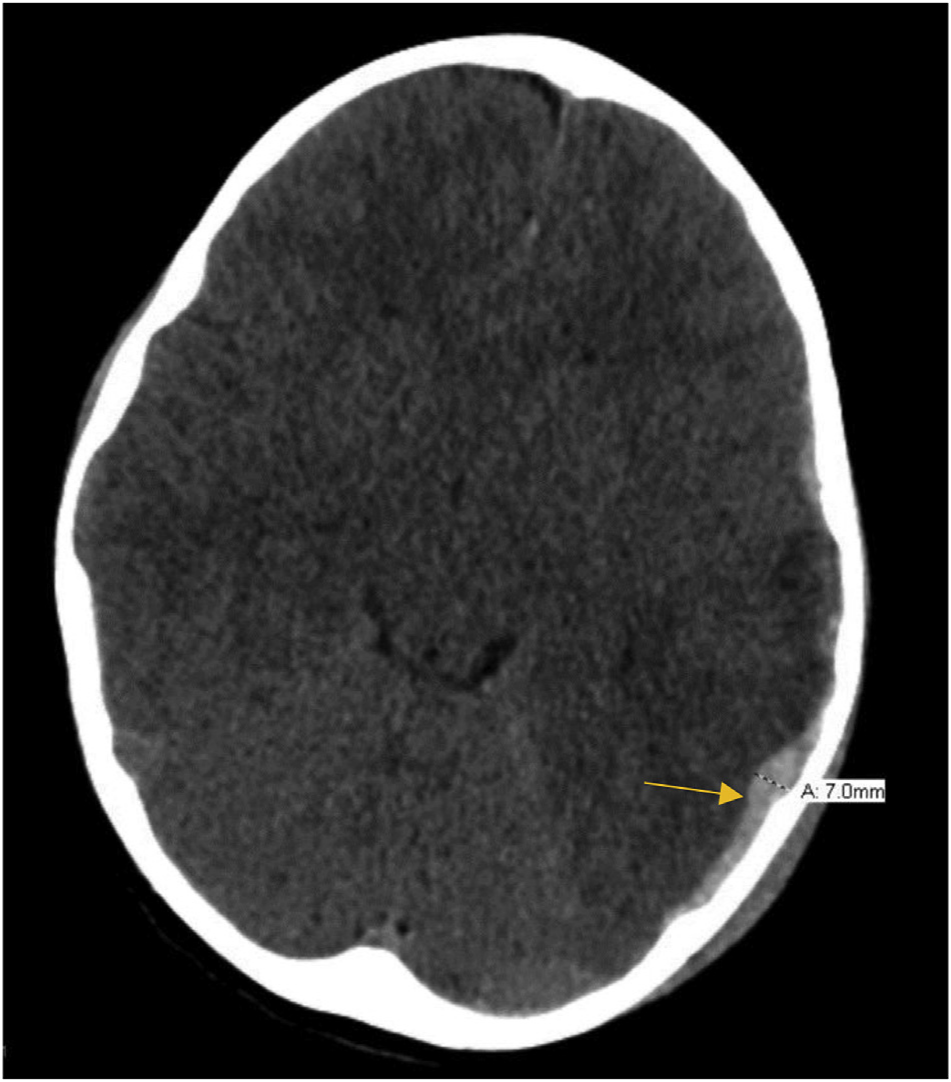
CT scan taken on second day of hospital admission after onset of bradycardic episodes. Results indicated expanding hematoma and development of midline shift.

**Fig. 3:**
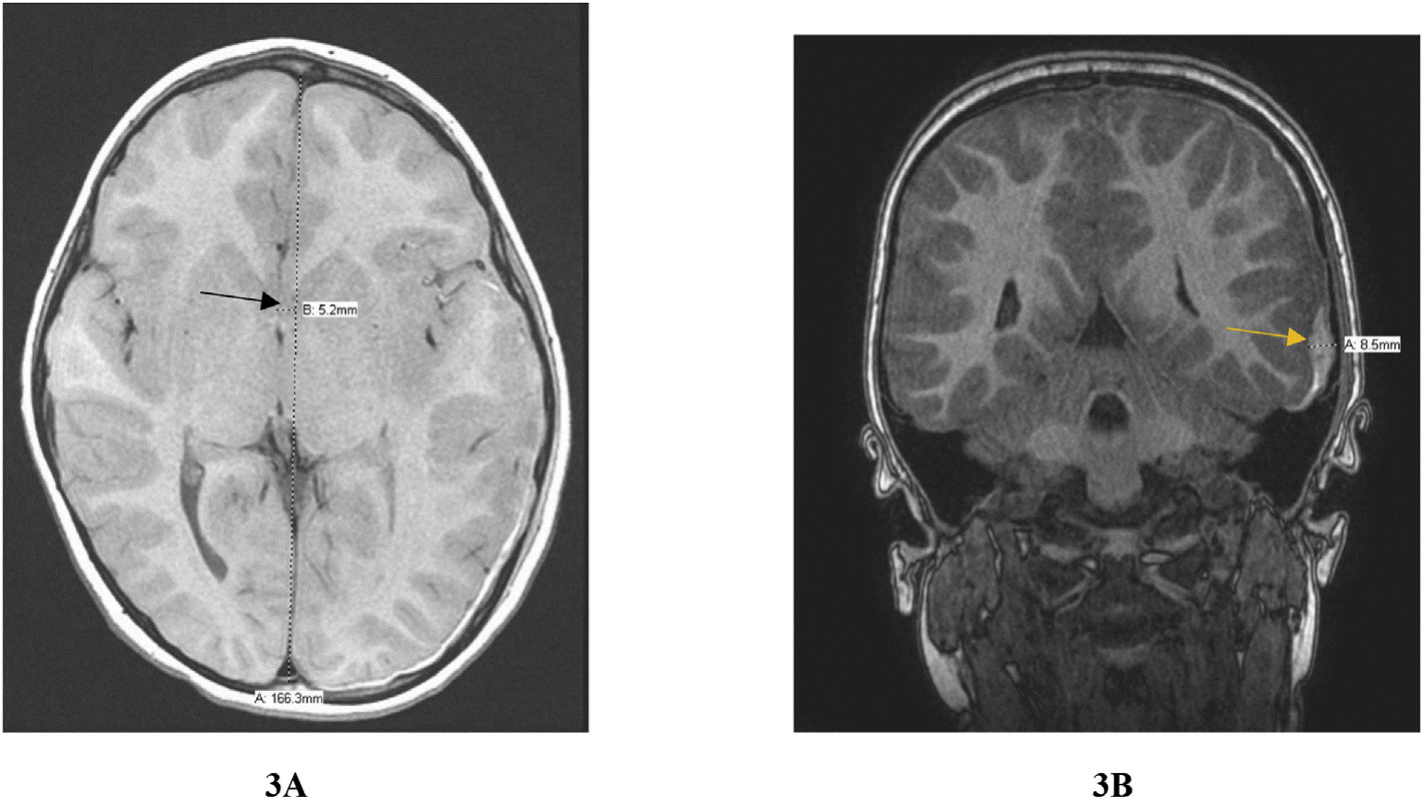
MRI scan completed on second day of hospital admission after bradycardic episodes. Image 3A highlights the midline shift (5.2mm) and Image 3B shows the expanding hematoma (8.5mm).

On day 1 post-injury, the patient still had significant nausea and emesis, and also complained of a frontal headache. Additionally, the patient began to develop episodes of bradycardia. These episodes were transient and self-resolving, with his heart rate decreasing to 30 bpm and saturation to 79% on room air. The patient remained alert and fully oriented during these episodes. A CT scan was ordered to assess any extension of the hematoma and increased intracranial pressure (Fig. 2). Results indicated temporal swelling and a 3mm midline shift. An MRI indicated that the hematoma had a maximum thickness of 8–9mm, and other small contusions were also noted. The MRI also showed a midline shift of approximately 5 mm (Fig. 3).

The patient was then started on a 3% saline infusion at a rate of 15 mL/h for 24 hours. This improved the patient's nausea and decreased the volume of emesis. After the infusion was stopped, the patient began having episodes of bradycardia, apnea, and desaturation while sleeping. The patient's monitor documented eleven episodes, but nursing staff claimed to have seen nearly 30 apneic episodes in one evening. The saline infusion was restarted that evening, in addition to levetiracetam (Keppra) and non-invasive ventilation while sleeping. During this time, the patient retained a GCS of 15.

**Fig. 4:**
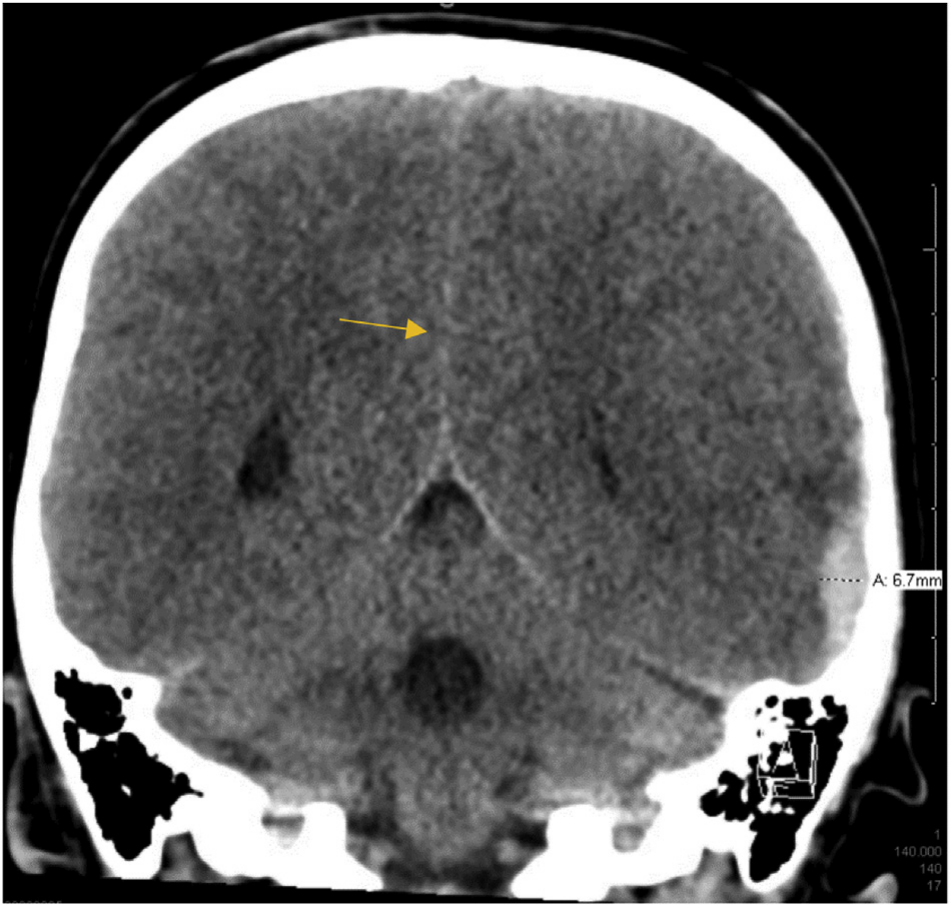
CT scan taken on seventh day of hospital admission indicates absence of midline shift.

The patient became less symptomatic while on the infusion, and further testing was performed. An EKG showed sinus bradycardia and an EEG was unremarkable. The next morning, the patient had apneic episodes with desaturation when sleeping. When awake, he was fully alert and oriented. He was given a bolus of 75mL of 3% saline and the infusion rate was increased to 20 mL/h. Initially his symptoms resolved, but his apneic episodes returned the next evening. His infusion rate was increased to 25 mL/h. A repeat CT scan indicated no midline shift (Fig. 4).

The next day, the patient was started on oral NaCl with the goal of de-escalating his infusions. He was steadily weaned off his infusions over the next several days, with discharge from the hospital two days later. Neuropsychology placed him on several restrictions. These ranged from limited school activity to decreased physical activity. While the patient had no altered mental status during this period, neuropsychological testing indicated long-term attention and executive function difficulties.

## 3 Discussion

TBIs have proven to be potentially devastating injuries, especially in pediatric patients. A national study from the Nationwide Emergency Department Sample (NEDS) reported that pediatric TBI-related ED visits have increased by about 30% in the US throughout the past decade [1]. In the United States each year, approximately 473,947 children visit the emergency department due to head trauma and of these, 35,000 are hospitalized and 2100 die as a direct result of their injury [5]. Additionally, the presenting symptoms in children with a TBI can be extremely variable and not necessarily similar to those of adults. In many cases, children are unaware that they are injured and therefore the unmasking of the symptoms through use of multiple diagnostic modalities must be done. These include, but are not limited to: GCS assessment, serial head CTs, frequent monitoring of ICP, and multiple developmentally-appropriate neurological exams, all of which can aid in the diagnosis of a pediatric TBI [6].

TBIs can be classified into primary and secondary injuries [7]. Primary injuries result from the initial impact or injury to the head which leads to epidural and subdural hematomas, as well as axonal shearing and cortical contusions [7]. Secondary injuries present hours to days following the insult and are the result of hypotension, hypoxia, cerebral edema and increased ICP of over 20 mmHg, and include non-mechanical damage that results from cellular disruption [5,7]. Our patient suffered from both primary and secondary injuries. His primary injury was seen through the skull fracture and subdural hematoma. However, the subsequent increase in ICP, as noted by the midline shift, may have been the major contributor to the episodes of desaturation and bradycardia.

As previously mentioned, pediatric patients have widely variable presentations and practitioners often use a combination of associated symptoms and imaging to fully assess the extent of the injury. CT is the most commonly used initial diagnostic test for TBIs due to its speed and ability to detect serious lesions that may require immediate intervention [7]. CT is specifically useful in detecting hemorrhages, cerebral edema, and bone fractures in TBIs [7]. Additionally, the use of MRI allows for complementary visualization of soft tissue structures in addition to CT imaging [7]. It has greater sensitivity in regards to axonal injury which is useful in prognostic determination. However, due to the increased time needed for imaging using an MRI, this is a secondary modality [7].

Common symptoms of traumatic brain injuries include lightheadedness, dizziness, nausea, vomiting, photophobia, and phonophobia [6]. Focal neurological deficits may also be evident upon exam such as fixed, dilated pupils, indicating a likely cerebral herniation or unilateral deficits signifying a midline shift and compression of a cerebral hemisphere. For detection of more global neurological deficiencies, the GCS may be used. The GCS aides in grading the severity of TBI and consists of three markers for scoring on a scale ranging from 3 (totally unresponsive) to 15 (maximally responsive) [8]. The GCS assessment components consist of: eye-opening response (1–4), best verbal response (1–5), and best motor response (1–6) [8]. A GCS score of 13–15 typically indicates a mild TBI, whereas, scores of 9–12 and less than or equal to 8 indicate moderate and severe TBI, respectively [7].

In our case, the patient initially presented with a closed skull fracture and small temporoparietal subdural hematoma. No localized neurological deficits or indications of herniation were found on physical exam, including assessment of cranial nerves and consciousness. His only indications of cerebral injury included nausea, vomiting, and diaphoresis. Because his GCS score remained at 15, only a mild injury was clinically indicated. Furthermore, the absence of neurological symptoms did not call for serial CT scans. However, the subsequent development of bradycardia as a result of increased ICP and parasympathetic response signaled a more severe injury than previously suspected. The CT and MRI demonstrated the expanding hematoma and increased ICP, due to the development of a midline shift. The TBI was more severe than initially thought, but the patient remained neurologically intact.

The next dilemma was to determine the appropriate course of management. While the guidelines for adult ICP management are well-established, there are only weak guidelines available for pediatric management [9]. Medical management and surgical decompression are classified as Level II and Level III recommendations, respectively [9]. The patient's initial hypertonic saline treatment led to rapid symptom resolution, and discontinuation of treatment led to symptom rebound. The subsequent initiation of hypertonic saline proved to be a successful treatment, with eventual resolution of the midline shift and de-escalation to an oral medication. It is unknown if surgical decompression would have yielded similar results, but in this case medical treatment was sufficient to relieve symptoms without the additional stress of surgery.

Long term sequelae that can occur in the pediatric population following a traumatic brain injury include: executive function disruption, inattention, memory lapses, insomnia and more [6]. Additionally, a study at the Department of Pediatrics at the University of Texas Health Science Center found that TBIs in children 6 years old and younger were associated with decreases in working memory and inhibitory control [5]. These findings are consistent with the rapid development hypothesis, which states that the areas of the brain that are in an increased rate of development during childhood will be particularly vulnerable to disruption by TBIs [5]. With our patient, the neuropsychiatric testing indicated there will be long term cognitive deficiencies, and he was given a schedule on how to ease back into school in anticipation of this. Even without the presentation of neurological symptoms acutely, the patient is expected to experience long-term effects from this injury. This work has been reported in line with the SCARE criteria [10].

## 4 Conclusion

Children may differ in their response to TBIs as some children's brains are more resilient to trauma than others, which affects their cognitive response. TBIs are usually associated with an alteration of mental status but this may not always be the case - continuous monitoring of pediatric patients until resolution of all symptoms regardless of GCS is important in successful recovery and prevention of secondary injuries from a TBI. Our patient is an example of the discrepancy that can sometimes present between clinical judgment based on external assessment and those acquired through imaging modalities.

## Ethical approval

NA.

## Sources of funding

This research did not receive any specific grant from funding agencies in the public, commercial, or not-for-profit sectors.

## Author contribution

**FA,** First author. Coordinated the design of this study, performed literature reviews, contributed in data collection from the preoperative, intraoperative and postoperative periods. Helped in writing the manuscript and provided proofreading of the report and gave its final form for submission.

**AD,** Co-author. Did literature reviews and contributed in collection of data from the preoperative, intraoperative and postoperative periods. Helped in writing the manuscript.

**CC,** Co-author. Provided editing and proofreading for this case.

**HD,** Co-author. Did literature reviews and contributed in collection of data from the preoperative, intraoperative and postoperative periods. Helped in writing the manuscript.

**G S D,** Co-author. Is the main care provider in this case. Provided feedback and proofreading for this case, and he approved it for submission.

All the authors had read and approved the final report.

## Conflicts of interest

None. This work has been reported in line with the SCARE criteria.

## Research registration number

NA.

## Guarantor

G S D.

## Consent

Written informed consent was obtained from the patient for publication of this case report and accompanying images. A copy of the written consent is available for review by the Editor-in-Chief of this journal on request. This case report doesn't have any identifying information about the patient, so patient's guardian approved publishing this case report. Any identifying information requires another consent from the patient's guardian.

## Provenance and peer review

Not commissioned, externally peer reviewed.

## Appendix A Supplementary data

SCARE Guideline Checklist SCARE Guideline Checklist
